# ﻿Complete mitochondrial genomes of the black corals *Alternatipathesmirabilis* Opresko & Molodtsova, 2021 and *Parantipatheslarix* (Esper, 1788) (Cnidaria, Anthozoa, Hexacorallia, Antipatharia, Schizopathidae)

**DOI:** 10.3897/zookeys.1196.116837

**Published:** 2024-03-22

**Authors:** Brendan A. Cruz, Anneau Cappelmann, Hope Chutjian, Jude C. Roman, Mason A. Reid, Jacob Wright, Aydanni D. Gonzalez, Taylor Keyman, Kierstin M. Griffith, Hannah J. Appiah-Madson, Daniel L. Distel, Vonda E. Hayes, Jim Drewery, D. Tye Pettay, Joseph L. Staton, Mercer R. Brugler

**Affiliations:** 1 Department of Natural Sciences, University of South Carolina Beaufort, 1100 Boundary St, Beaufort, SC 29902, USA University of South Carolina Beaufort Beaufort United States of America; 2 Ocean Genome Legacy Center, Northeastern University, 430 Nahant Road, Nahant, MA 01908, USA Northeastern University Nahant United States of America; 3 Department of Fisheries & Oceans Canada, Northwest Atlantic Fisheries Centre, 80 East White Hills Road, St. John’s, Newfoundland & Labrador, A1C 5X1, Canada Northwest Atlantic Fisheries Centre Newfoundland & Labrador Canada; 4 Marine Directorate of Scottish Government, Marine Laboratory, 375 Victoria Road, Aberdeen AB11 9DB, Scotland, UK Marine Directorate of Scottish Government, Marine Laboratory Aberdeen United Kingdom; 5 Division of Invertebrate Zoology, American Museum of Natural History, Central Park West at 79th Street, New York, NY 10024, USA American Museum of Natural History New York United States of America; 6 Department of Invertebrate Zoology, National Museum of Natural History, Smithsonian Institution, 10th St. & Constitution Ave. NW, Washington, DC 20560, USA National Museum of Natural History, Smithsonian Institution Washington United States of America

**Keywords:** Antipatharian, genome skimming, holotype, intraspecific variation, Mitofinder, *Parantipathes*, *Stauropathesarctica*

## Abstract

We describe the complete mitogenomes of the black corals *Alternatipathesmirabilis* Opresko & Molodtsova, 2021 and *Parantipatheslarix* (Esper, 1790) (Cnidaria, Anthozoa, Hexacorallia, Antipatharia, Schizopathidae). The analysed specimens include the holotype of *Alternatipathesmirabilis*, collected from Derickson Seamount (North Pacific Ocean; Gulf of Alaska) at 4,685 m depth and a potential topotype of *Parantipatheslarix*, collected from Secca dei Candelieri (Mediterranean Sea; Tyrrhenian Sea; Salerno Gulf; Italy) at 131 m depth. We also assemble, annotate and make available nine additional black coral mitogenomes that were included in a recent phylogeny (Quattrini et al. 2023b), but not made easily accessible on GenBank. This is the first study to present and compare two mitogenomes from the same species of black coral (*Stauropathesarctica* (Lütken, 1871)) and, thus, place minimum boundaries on the expected level of intraspecific variation at the mitogenome level. We also compare interspecific variation at the mitogenome-level across five different specimens of *Parantipathes* Brook, 1889 (representing at least two different species) from the NE Atlantic and Mediterranean Sea.

## ﻿Introduction

Black corals (Cnidaria, Anthozoa, Hexacorallia, Antipatharia) are found in all oceans and hold the record for the deepest (*Schizopathesaffinis* Brook, 1889 at 8,900 m; Molodtsova (2006)) and longest-lived (*Leiopathesglaberrima* (Esper, 1792) at 4,265 years; Roark et al. (2009)) coral and serve as underwater hosts for a diverse and staggering number of epibionts (Love et al. 2007). Black corals have historically been considered a deep-water group; however, only 31.57% of the 285 currently-described species occur at depths greater than 800 m (Molodtsova et al. 2022, 2023). While the black coral community continues to wait for the first antipatharian nuclear genome, black coral mitogenomics is gaining in popularity due to the ease of bioinformatically extracting whole mitogenomes from genome-skimming data (Quattrini et al. 2023a), its informativeness, cost-effectiveness and the availability of comparative data (Brugler and France 2007; Sinniger and Pawlowski 2009; Kayal et al. 2013; Figueroa et al. 2019; Barrett et al. 2020; Asorey et al. 2021; Bledsoe-Becerra et al. 2022; Feng et al. 2023; Quattrini et al. 2023a, b; Ramos et al. 2023). Herein, we describe two additional black coral mitogenomes (*Alternatipathesmirabilis* Opresko & Molodtsova, 2021 and *Parantipatheslarix* (Esper, 1790)), both from the family Schizopathidae and present them in a phylogenetic context. We analysed the holotype of *Alternatipathesmirabilis*, collected from Derickson Seamount (North Pacific Ocean; Gulf of Alaska) at 4,685 m depth and a potential topotype of *Parantipatheslarix*, collected from Secca dei Candelieri (Mediterranean Sea; Tyrrhenian Sea; Salerno Gulf; Italy) at 131 m depth. According to Esper (1792), the type locality of *P.larix* is in the “ocean near Naples,” which is adjacent to the Gulf of Salerno.

The genus *Alternatipathes* was established by Molodtsova and Opresko (2017) with the type species of the genus as *Umbellapathesbipinnata* Opresko, 2005 (Opresko and Molodtsova 2021). Species assigned to the genus include *Umbellapathesbipinnata*, *Bathypathesalternata* Brook, 1889, *Alternatipathesvenusta* Opresko & Wagner, 2020 and *Alternatipathesmirabilis*. *Alternatipathesmirabilis* is only known from a single specimen, which we analysed herein. The species name (*mirabilis*) is derived from Latin meaning “wonderful or strange.” The genus is broadly distributed in the Pacific, Indian, Atlantic and Southern Ocean basins at depths usually exceeding 2,500 m and often greater than 4,000 m (Opresko and Molodtsova 2021). DNA analysis of mitochondrial *nad5-nad1* from the holotype suggested a close relationship to *Schizopathes* Brook, 1889 (Chery et al. 2018); however, the full mitogenome of *Schizopathes* is not currently available to test this hypothesis more robustly. The genus *Parantipathes* was established by Brook (1889) with the type species of the genus as *Antipatheslarix* Esper, 1790 (Opresko and Baron-Szabo 2001). In terms of its distribution, this species is only known from the Mediterranean Sea and eastern Atlantic Ocean.

In addition to describing the mitogenomes of *Alternatipathesmirabilis* and *Parantipatheslarix*, we also assembled, annotated and made available nine additional black coral mitogenomes that were included in a recent phylogeny (Quattrini et al. 2023b), but not made easily accessible on GenBank (i.e. mtDNA reads are embedded in non-annotated bulk Illumina whole genome shotgun fastq files). The taxa include *Acanthopathesthyoides* (Pourtalès, 1880) (USNM1288453), *Aphanipathespedata* (Gray, 1857) (USNM1288458), *Bathypathesalaskensis* Opresko & Molodtsova, 2021 (USNM1288462), *Elatopathesabietina* (Pourtalès, 1874) (USNM1288451), *Parantipathes* sp. (MSS29), *Stauropathesarctica* (Lütken, 1871) (DFONL ID #4089; Canadian Museum of Nature catalogue #CMNI 2023-0258), *Stauropathes* sp. Opresko, 2002 (USNM1404493), *Telopathesmagna* MacIsaac & Best, 2013 (MacIsaac et al. 2013) (USNM1204049) and *Umbellapathes* sp. Opresko, 2005 (USNM1404092).

Herein, we also compare two mitogenomes from the same species of black coral (*Stauropathesarctica*) and determine the expected level of intraspecific variation at the mitogenome level, which has not been done previously. We compare the results of this intraspecific comparison to the unexpectedly low mitogenome-level variation found within the trigeneric complex (*Dendrobathypathes*, *Lillipathes* and *Parantipathes* from the eastern North Pacific; Bledsoe-Becerra et al. (2022)). We also compare interspecific variation at the mitogenome-level across five different specimens of *Parantipathes* (representing at least two different species) from the northeast Atlantic and Mediterranean Sea.

## ﻿Materials and methods

### ﻿Specimen collection and species identification

The holotype of *Alternatipathesmirabilis* Opresko & Molodtsova, 2021 (USNM1070972) was collected by Dr. Amy Baco-Taylor on 20 July 2004, from Derickson Seamount (North Pacific Ocean; Gulf of Alaska; Station # JD-093) at 4,685 m depth using the Jason II ROV (Latitude, Longitude: 53.0419, -161.183). The holotype of *A.mirabilis* was deposited into the black coral collection at the Smithsonian Institution’s National Museum of Natural History (NMNH). Specimens accessioned into the SI NMNH’s Invertebrate Zoology collection are freely available to researchers to access and study. *A.mirabilis* was identified by Drs. Dennis Opresko and Tina Molodtsova, the leading authorities on black coral taxonomy and systematics. *Parantipatheslarix* (Esper, 1788) (USNM1280881) was collected in July 2012 from Secca dei Candelieri (Mediterranean Sea; Tyrrhenian Sea; Salerno Gulf; Italy) at 131 m depth (Latitude, Longitude: 40.0744, 15.8765). *P.larix* was also deposited into the SI NMNH’s Invertebrate Zoology collection. *P.larix* was identified by Dr. Marzia Bo of the Universita di Genova in Italy, also an authority on black coral taxonomy and systematics.

### ﻿Specimen preparation and sequencing

Tissues from *Alternatipathesmirabilis* (OGL-E27108; USNM1070972) and *Parantipatheslarix* (OGL-E27184; USNM1280881) were initially stored in 95% ethanol. DNA was isolated from these samples using a modified CTAB extraction protocol (France et al. 1996). Specifically, tissue samples were incubated in 750 μl of 2X-CTAB with 50 μl Proteinase K (Qiagen, Hilden, Germany) overnight before digestion at 56 °C for 3 hours. Ceramic beads (200 μl, 0.1 mm) were added to each sample and tubes were placed in a BeadBug microtube homogeniser (Benchmark Scientific, South Plainfield, NJ, USA) for two 30 second intervals at 2,800 rpm. Next, particulate material was precipitated by centrifugation at 17K RCF (Relative Centrifugal Force or g-force) for 5 minutes and the supernatants were transferred to new tubes with 750 μl of -20 °C chloroform, vortexed until cloudy and phases were separated by centrifugation at 17K RCF for 10 minutes. Supernatants were then transferred to tubes with 750 μl of -20 °C absolute ethanol, inverted and phases were separated by centrifugation at 17K RCF for 5 minutes. Supernatants were discarded and precipitated DNA was washed with 750 μl of 70% ethanol and then pelleted by centrifugation at 17K RCF for 5 min. Supernatants were again discarded and pellets were dried using a Savant DNA 120 Speedvac Concentrator (Thermo Scientific, Waltham, MA, USA) before suspension in 50 μl of Buffer AE (Qiagen, Hilden, Germany). DNA extracts were subsequently treated with RNase A and purified using a Zymo Research DNA Clean & Concentrator (Irvine, CA, USA). To visualise DNA, 2 μl of each extract was loaded on to a horizontal slab gel (1% agarose, 1X TAE buffer containing 1% Biotium GelRed nucleic acid gel stain; Freemont, CA, USA) and separated at approximately 175 V for 5 min then 130 V for 30 min and visualised using a Bio-Rad Gel Doc XR + Molecular Imager and Image Lab software (Hercules, CA, USA). To quantify DNA present in each extract, 5 μl of each sample was analysed using a Promega QuantiFluor ONE dsDNA System with a Quantus Fluorometer (Madison, WI, USA). DNA extractions were sent to the New York Genome Center for whole genome shotgun (WGS) sequencing on an Illumina HiSeqX (2x150 bp). Library preparation utilised a TruSeq PCR-free kit (450 bp).

### ﻿Bioinformatics

Mitochondrial genomes were bioinformatically extracted from the WGS runs using MitoFinder v.1.4 (Allio et al. 2020). MitoFinder employed MEGAHIT v.3.0 (Li et al. 2015) for mitogenome assembly and tRNAscan-SE (Chan and Lowe 2019) for tRNA annotation. The following command was used to run MitoFinder on an iMac: ./mitofinder --megahit --override --new-genes -j [file name] -1 [left_reads.fastq.gz] -2 [right_reads.fastq.gz] -r [genbank_reference.gb] -o [genetic_code] -p [threads] -m [memory] -t trnascan. *Stichopathesluetkeni* (GenBank Accession # NC_018377) was used as the reference and translation table 4 (Mold, Protozoan and Coelenterate Mitochondrial Code and the Mycoplasma/Spiroplasma Code) was used as the genetic code. Newly-assembled mitogenomes were annotated using the MITOS Web Server (Bernt et al. 2013).

### ﻿Phylogenetic analysis

**Figure 1.**
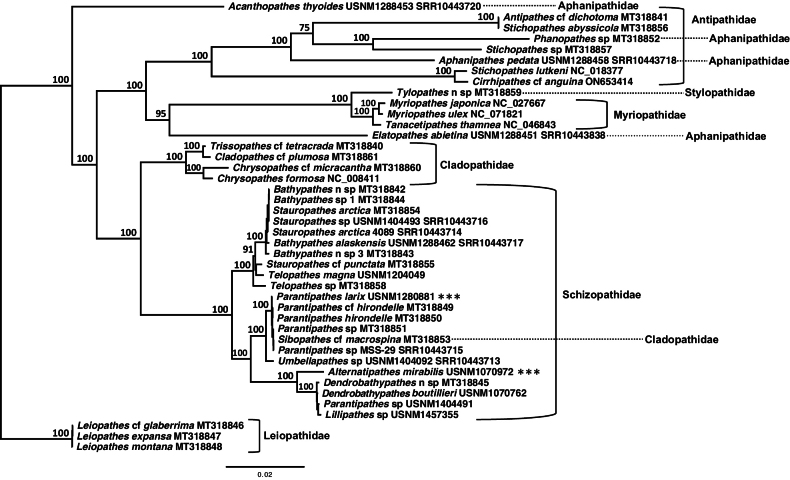
Maximum Likelihood phylogenetic tree, based on 13 protein-coding genes and two ribosomal RNAs (42 taxa and 16,416 sites). The mitogenomes of *Alternatipathesmirabilis* (USNM1070972; OR398473) and *Parantipatheslarix* (USNM1280881; OR398474) are indicated with three asterisks. The families Aphanipathidae and Cladopathidae are polyphyletic with representatives indicated with a horizontal dotted line. The tree is rooted internally to the Leiopathidae. Node support values are based on 1,000 ultrafast bootstrap replicates. Species IDs are followed by museum voucher codes (e.g. USNM) and/or GenBank accession numbers (e.g. MT, NC, ON or SRR).

The newly-obtained mitogenomes of *Alternatipathesmirabilis* (USNM1070972; GenBank Accession Number OR398473) and *Parantipatheslarix* (USNM1280881; GenBank Accession Number OR398474) were added to the phylogeny presented in Bledsoe-Becerra et al. (2022) that contained 29 mitogenomes. We then assembled, annotated and added nine black coral mitogenomes that were included in a recent phylogeny (Quattrini et al. 2023b), but not made easily accessible on GenBank. We also included two newly-released black coral mitogenomes: *Myriopathesulex* (Ellis & Solander, 1786) (NC_071821) and Cirrhipathescf.anguina (Dana, 1846) (ON653414; Shizuru et al. (2024)) for a total of 42 taxa. Each of the 13 protein-coding genes (*cox1-3*, *nad1-6*, *nad4L*, *atp6*, *atp8* and *cytb*) and two ribosomal RNAs (12S and 16S) from all 42 mitogenomes were placed in individual AliView v.1.23 (Larsson 2014) files, individually aligned using MAFFT LINS-i v.7 (Katoh et al. 2019) and subsequently concatenated into a single file using Seqotron v.1.0.1 (Fourment and Holmes 2016), treating the mitogenome as a single locus. Significant length variation was encountered within each of the 18 intergenic regions (IGRs) across the seven families, resulting in ambiguous alignments within these regions; thus, IGRs were not considered. The final dataset consisted of 42 taxa and 16,416 sites (alignment available upon request to co-author Brugler). The Akaike Information Criterion within jModelTest v.2.1.10 (Guindon and Gascuel 2003; Darriba et al. 2012) selected the GTR + I + G model of sequence evolution (p-inv: 0.4670; gamma: 1.0920). XSEDE on the CIPRES Science Gateway v.3.3 (Miller et al. 2011) was used to construct a Maximum Likelihood phylogeny using IQ-Tree v.2.2.2.5 with the GTR+I+G model of sequence evolution, a BioNJ starting tree and 1,000 ultrafast bootstrap replicates (Hoang et al. 2018; Minh et al. 2020). The resulting phylogenetic tree was visualised using FigTree v.1.4.4 (by Andrew Rambaut; https://github.com/rambaut/figtree/releases). The ML tree (Fig. 1) was rooted internally to the Leiopathidae. This decision was based on: 1) the mitogenome-based phylogeny presented in Barrett et al. (2020) that included nine hexacoral outgroups (4 actiniarians, 3 zoantharians, 1 scleractinian and 1 corallimorpharian) and 2) a time-calibrated phylogeny by Horowitz et al. (2023), based on target-capture enrichment of 2,380 ultraconserved elements and exonic loci from 83 species of black coral and nine outgroups, both of which recovered the Leiopathidae as an early branching, monophyletic group sister to all other antipatharian families (but see DeSalle et al. (2023)). The number of variable sites (or single nucleotide polymorphisms; SNPs) and pairwise distance estimates were calculated using MEGA X (Kumar et al. 2018; Stecher et al. 2020) and included the Kimura 2-Parameter model (K2P), uniform rates amongst sites and pairwise deletion of gaps/missing data.

## ﻿Results

The *Alternatipathesmirabilis* mitogenome (OR398473) is 17,632 bp in length and contains the typical 13 protein-coding genes (*cox1-3*, *nad1-6*, *nad4L*, *atp6*, *atp8* and *cytb*), two ribosomal RNAs (12S and 16S) and two transfer RNAs (Met and Trp). Intergenic regions account for 8.72% (1,538 bp) of the mitogenome, with the longest IGR between *nad5* (5’) and *nad1* (365 bp; Table 1). The *Parantipatheslarix* mitogenome (OR398474) is 17,734 bp in length and contains the typical 13 protein-coding genes (*cox1-3*, *nad1-6*, *nad4L*, *atp6*, *atp8* and *cytb*), two ribosomal RNAs (12S and 16S) and two transfer RNAs (Met and Trp). Intergenic regions account for 9.41% (1,669 bp) of the mitogenome, with the longest IGR between *nad5* (5’) and *nad1* (367 bp; Table 1). Both *A.mirabilis* and *P.larix* have the typical black coral mitochondrial gene order; thus, to date, one unique gene order has been observed within the Order Antipatharia. Base composition was similar between *A.mirabilis* (A: 5841, T: 4701, G: 3180, C: 3910) and *P.larix* (A: 5895, T: 4708, G: 3212, C: 3919) and both mitogenomes are AT-rich (59.79% each).

**Table 1.** Lengths of protein-coding genes, ribosomal RNAs, transfer RNAs and intergenic regions (IGRs) within the *Alternatipathesmirabilis* (17,632 bp; OR398473) and *Parantipatheslarix* (17,734 bp; OR398474) mitogenomes.

Gene	*Parantipatheslarix*	*Alternatipathesmirabilis*
**12S**	1141	1141
**IGR**	197	197
***nad2***	1518	1518
**IGR**	19	19
**tRNA Trp**	70	70
**IGR**	27	27
***nad5*-3**’	1131	1131
**IGR**	115	115
***nad3***	357	357
**IGR**	48	32
***nad1***	984	984
**IGR**	367	365
***nad5*-5**’	708	708
**IGR**	108	46
***atp6***	699	699
**IGR**	82	82
***atp8***	213	213
**IGR**	24	24
***nad6***	564	564
**IGR**	104	87
***nad4***	1476	1476
**IGR**	61	61
***cox2***	750	750
**IGR**	82	68
***nad4L***	300	300
**IGR**	92	92
***cox1***	1590	1590
**IGR**	34	34
***cox3***	789	789
**IGR**	96	74
**16S**	2561	2590
**IGR**	64	74
**tRNA Met**	71	71
**IGR**	49	40
***cytb***	1143	1143
**IGR**	100	101

After assembling nine mitogenomes that were included in a phylogeny in Quattrini et al. (2023b), mitogenome sizes ranged from 17,699 (*Bathypathesalaskensis* USNM1288462) to 20,066 bp (*Acanthopathesthyoides* USNM1288453). Quattrini et al. (2023b) simply uploaded Illumina fastq files and each of the 13 protein-coding genes individually, but did not upload the two ribosomal RNAs (12S and 16S), two transfer RNAs (Met and Trp) or any of the intergenic regions. To make the data more easily accessible, we pulled data from the Illumina fastq files to assemble and annotate full mitogenomes. Complete nucleotide sequence data are now available in the Third Party Annotation (TPA) section of the DDBJ/ENA/GenBank databases under the following accession numbers: BK063761 (*Acanthopathesthyoides*; USNM1288453), BK063759 (*Aphanipathespedata*; USNM1288458), BK063764 (*Bathypathesalaskensis*; USNM1288462), BK063760 (*Elatopathesabietina*; USNM1288451), BK063757 (*Parantipathes* sp.; MSS29), BK063763 (*Stauropathesarctica*; DFONL ID #4089; Canadian Museum of Nature catalogue #CMNI 2023-0258), BK063762 (*Stauropathes* sp.; USNM1404493), OR398475 (*Telopathesmagna*; USNM1204049) and BK063758 (*Umbellapathes* sp.; USNM1404092). The two newly-released black coral mitogenomes ranged in size from 17,711 bp (*Myriopathesulex*NC_071821 [OP104910]; released 3 April 2023) to 20,452 bp (Cirrhipathescf.anguinaON653414; released 24 December 2022; Shizuru et al. (2024)). *Elatopathesabietina* (BK063760) and *Myriopathesulex* (NC_071821) were the only taxa that contained a LAGLI-DADG homing endonuclease in the *cox1* gene (Beagley et al. 1996).

## ﻿Discussion

The Maximum Likelihood phylogeny (Fig. 1), consisting of 42 taxa and 16,416 sites, largely mirrors the phylogeny presented in Brugler et al. (2013) that was based on three mitochondrial gene regions (*igrW*, *igrN* and *cox3*-*cox1*); however, the mitogenome-based phylogeny, presented here, yields greater bootstrap support. In our new mitogenome-based phylogeny, the holotype of *Alternatipathesmirabilis* is sister to a clade containing *Dendrobathypathes* Opresko, 2002, *Parantipathes* (from the North Pacific Ocean) and *Lillipathes* Opresko, 2002 (bootstrap support: 100), while a putative topotype of *Parantipatheslarix* is placed within a clade containing additional *Parantipathes* (all from the Northeast Atlantic), *Sibopathes* van Pesch, 1914 and *Umbellapathes* (bootstrap support: 100). *Sibopathes* is currently classified in the family Cladopathidae yet falls within the Schizopathidae in our analyses. However, any potential reclassification of this genus should include data from the type specimen of *Sibopathes*. These data were not available at the time of this analysis.

According to our analyses, the family Aphanipathidae is polyphyletic with representatives forming a group sister to the majority of antipatharians (*Acanthopathesthyoides* USNM1288453; bootstrap support: 100), sister to the Myriopathidae (*Elatopathesabietina* USNM1288451; bootstrap support: 95) or sister to different representatives of the Antipathidae (*Aphanipathespedata* USNM1288458 and *Phanopathes* sp. Opresko, 2004 MT318852; bootstrap support: 100; taxon sampling within the Antipathidae is very limited as our phylogeny only includes five of 122+ species within the family).

In Brugler et al. (2013), *Acanthopathesthyoides* (USNM1288453) and *Elatopathesabietina* (USNM1288451) were considered “wandering taxa” as their phylogenetic relationship shifted depending on the dataset or tree-building algorithm. It appears that our new mitogenome-based phylogeny has stabilised their position and revealed more strongly-supported phylogenetic affiliations for both taxa.

Only one representative from the family Stylopathidae was included in the phylogeny (*Tylopathes* sp. nov. Brook, 1889 MT318859) and is sister to the Myriopathidae (bootstrap support: 100). Any potential reclassification of these genera within the Myriopathidae will require sequence data from the remaining genera within the Stylopathidae (*Stylopathes* Opresko, 2006 and *Triadopathes* Opresko, 2006). These data were not available at the time of this analysis.

To our knowledge, this study is the first to compare two mitogenomes from the same species of black coral (*Stauropathesarctica*MT318854 and CMNI 2023-0258) and thus we can, for the first time, place lower limits on the expected level of intraspecific variation at the mitogenome level. Both mitogenomes are 17,700 bp in length and a comparison revealed 12 SNPs (K2P distance: 0.0678%). *Stauropathesarctica* (MT318854) was collected at 1,446 m depth from North Porcupine Bank (NE Atlantic; Irish Margin). *Stauropathesarctica* (CMNI 2023-0258) was collected at 600 m depth from Treworgie Canyon (NW Atlantic; Grand Banks of Newfoundland). Bledsoe-Becerra et al. (2022) compared the mitogenomes of the trigeneric complex (*Dendrobathypathes*, *Lillipathes* and *Parantipathes* from the eastern North Pacific) and only found 32 SNPs across 17,687 bp. Pairwise comparisons revealed 18 (*Dendrobathypathes* and *Parantipathes*) and 23 (*Lillipathes* and *Parantipathes*; *Lillipathes* and *Dendrobathypathes*) SNPs. If future mitogenomic studies show that approximately 12 SNPs are typical of intraspecific comparisons within the Antipatharia, then 18 and 23 SNPs may be indicative of interspecific variation and, thus, *Dendrobathypathes*, *Lillipathes* and *Parantipathes* (from the eastern North Pacific) could be consolidated into a single genus. However, a black coral nuclear genome is not available at this time, which could fundamentally change our understanding of species relationships within this group. Therefore, a major consolidation of multiple genera is not advised until nuclear genomes are also sequenced and analysed. It is also important to note that the mitogenome-level comparisons noted above (for *Stauropathes*, *Dendrobathypathes*, *Lillipathes* and *Parantipathes*) are all for taxa within the family Schizopathidae and, thus, variation within, or thresholds between, other families may differ given their different evolutionary histories. As per Horowitz et al. (2023), 95% of extant black corals were recovered in two distinct clades that diverged ~ 295 million years ago (during the Carboniferous-Permian) on the continental slope. The first clade contained members of the Antipathidae, Aphanipathidae, Myriopathidae and Stylopathidae with crown node at 242 My; these taxa largely stayed on the slope or moved up on to the shelf. The second clade contained members of the Schizopathidae and Cladopathidae with crown node at 202 My; these taxa are largely found at slope and abyssal depths.

We also had the unique opportunity to compare interspecific variation at the mitogenome-level across five different specimens of *Parantipathes* from the NE Atlantic and Mediterranean Sea (Parantipathescf.hirondelleMT318849; *Parantipatheshirondelle* Molodtsova, 2006 MT318850; *Parantipatheslarix* USNM1280881; *Parantipathes* sp. MSS-29; *Parantipathes* sp. MT318851). We also included Sibopathescf.macrospina (MT318853) in this analysis as it groups phylogenetically amongst these five *Parantipathes*. All six mitogenomes are 17,734 bp in length and a comparison revealed only 18 SNPs (K2P distances ranged from 0.00564% [Sibopathescf.macrospinaMT318853 vs. *Parantipathes* sp. MT318851 and *Parantipathes* sp. MSS-29 and Parantipathescf.hirondelleMT318849 vs. *Parantipatheshirondelle*MT318850] to 0.0843% [*Parantipatheslarix* USNM1280881 vs. *Parantipathes* sp. MSS-29]). These results also support consolidating *Dendrobathypathes*, *Lillipathes* and *Parantipathes* (from the eastern North Pacific) into a single genus. Again, obtaining sequence data from the type specimen of *Sibopathes* will be necessary prior to the potential reclassification of this genus.

We encourage future black coral mitogenomic studies to focus on obtaining mitogenomes from type species (where possible) and continue to fill in missing taxonomic gaps, particularly in the Antipathidae, Aphanipathidae, Myriopathidae and Stylopathidae.

While morphological characteristics are the gold standard for delineating relationships amongst organisms, the combined use of morphology and genetics is a powerful combination to better understand evolutionary relationships (e.g. Wagner et al. (2010); Horowitz et al. (2020)). In fact, many fields must entirely rely on genetics to characterise diversity at the family level and below because morphology is lacking (e.g. Blank and Trench (1985); LaJeunesse et al. (2014)) and/or morphological characteristics are problematic (e.g. Pinzón and Lajeunesse (2011); Pinzón et al. (2013); Rodríguez et al. (2014); Bledsoe-Becerra et al. (2022); Opresko et al. (2022); Molodtsova et al. (2023)). Based on the data presented here and clear ambiguities created when using morphological characteristics of black coral, we strongly advocate that the black coral community preferentially use diversity at the molecular level to delineate evolutionary relatedness between groups and morphology only be used to support relationships revealed by molecular analyses. We also urge the International Commission on Zoological Nomenclature (ICZN), which is responsible for producing the International Code of Zoological Nomenclature, to incorporate robust molecular comparisons into species descriptions to account for instances where morphology fails.

## ﻿Additional information

### Author ORCIDs

Brendan A. Cruz https://orcid.org/0009-0008-4422-6489

Anneau Cappelmann https://orcid.org/0009-0007-8700-5726

Hope Chutjian https://orcid.org/0009-0008-5821-9335

Jude C. Roman https://orcid.org/0009-0002-9297-8008

Mason A. Reid https://orcid.org/0009-0009-6794-3947

Jacob Wright https://orcid.org/0009-0007-3743-6181

Aydanni D. Gonzalez https://orcid.org/0009-0007-7049-1019

Taylor Keyman https://orcid.org/0009-0006-0844-8485

Kierstin M. Griffith https://orcid.org/0009-0003-6800-4091

Hannah J. Appiah-Madson https://orcid.org/0000-0001-8408-7729

Daniel L. Distel https://orcid.org/0000-0002-3860-194X

Vonda E. Hayes https://orcid.org/0000-0001-8153-5629

Jim Drewery https://orcid.org/0000-0003-4308-1798

D. Tye Pettay https://orcid.org/0000-0002-2060-3226

Joseph L. Staton https://orcid.org/0009-0002-8695-5563

Mercer R. Brugler https://orcid.org/0000-0003-3676-1226

### Data availability

Mitogenomic data are available in GenBank under accession numbers OR398473 (*Alternatipathesmirabilis* USNM1070972), OR398474 (*Parantipatheslarix* USNM1280881), BK063761 (*Acanthopathesthyoides* USNM1288453), BK063759 (*Aphanipathespedata* USNM1288458), BK063764 (*Bathypathesalaskensis* USNM1288462), BK063760 (*Elatopathesabietina* USNM1288451), BK063757 (*Parantipathes* sp. MSS29), BK063763 (*Stauropathesarctica* CMNI 2023-0258), BK063762 (*Stauropathes* sp. USNM1404493), OR398475 (*Telopathesmagna* USNM1204049) and BK063758 (*Umbellapathes* sp. USNM1404092). The phylogenetic tree can be found on figshare: https://doi.org/10.6084/m9.figshare.25130414.
